# The intracellular visualization of exogenous DNA in fluorescence microscopy

**DOI:** 10.1007/s13346-024-01563-4

**Published:** 2024-03-25

**Authors:** Christina Greitens, Jean-Christophe Leroux, Michael Burger

**Affiliations:** Institute of Pharmaceutical Sciences, Department of Chemistry and Applied Biosciences, ETH Zurich, 8093 Zurich, Switzerland

**Keywords:** Gene delivery, Non-viral vectors, DNA visualization, Fluorescence microscopy, Labeled DNA, DNA-binding proteins, Fluorescence in situ hybridization

## Abstract

In the development of non-viral gene delivery vectors, it is essential to reliably localize and quantify transfected DNA inside the cell. To track DNA, fluorescence microscopy methods are commonly applied. These mostly rely on fluorescently labeled DNA, DNA binding proteins fused to a fluorescent protein, or fluorescence in situ hybridization (FISH). In addition, co-stainings are often used to determine the colocalization of the DNA in specific cellular compartments, such as the endolysosomes or the nucleus. We provide an overview of these DNA tracking methods, advice on how they should be combined, and indicate which co-stainings or additional methods are required to draw precise conclusions from a DNA tracking experiment. Some emphasis is given to the localization of exogenous DNA inside the nucleus, which is the last step of DNA delivery. We argue that suitable tools which allow for the nuclear detection of faint signals are still missing, hampering the rational development of more efficient non-viral transfection systems.

**Graphical abstract:**

The intracellular visualization of exogenous DNA in fluorescence microscopy. Created with BioRender.com.

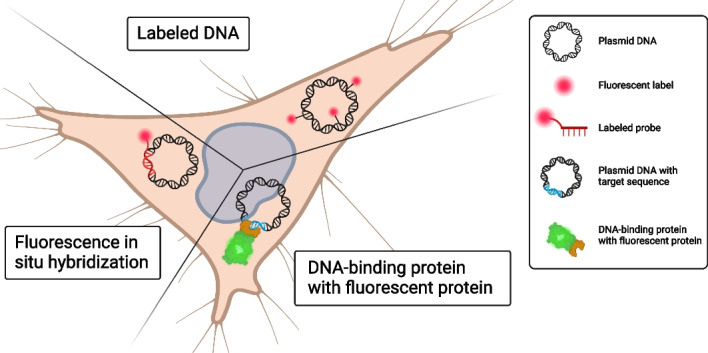

**Supplementary Information:**

The online version contains supplementary material available at 10.1007/s13346-024-01563-4.

## Introduction

Gene therapy comprises a wide array of applications that hold immense promise for treating severe diseases and manipulating living cells as well as whole organisms. Over several decades, extensive efforts have been made to develop transport vehicles for DNA. The delivered DNA can be either a complete gene or a short oligonucleotide. The challenge is to bring the DNA in vivo into cells where it has to be directed to its intracellular target site (e.g. nucleus). One common approach for gene delivery involves the use of modified viruses, some of which have evolved to navigate through the plasma membrane, traverse the cytoplasm, and ultimately release their DNA cargo directly into the nucleus, where transcription occurs [1, 2]. Alternatively, non-viral systems employing polymers, lipids, or proteins have been explored as synthetic DNA vehicles, as recently reviewed by Zu and Gao [3] and are summarized in Table 1. Unfortunately, non-viral systems suffer from inferior efficiency compared to their viral counterparts, and their mode of action on the cellular level is not yet fully understood. However, the demand for non-viral vectors is increasing as they promise a better safety profile, are cheaper to manufacture, less immunogenic, and are less restricted in their gene packaging capacity [3, 4].

**Table 1:** Non-viral gene delivery systems

**Delivery system**	**Example**	**Advantages**	**Disadvantages**	**Ref.**
Polymers	Poly(ethyleneimine) (PEI)	• High loading capacity • Simplicity	• Toxicity • Low specificity • Low serum tolerance	[5, 6]
Lipids	Lipofectamine (in vitro): (2,3‐dioleoyloxy‐N‐[2(sperminecarboxamido)ethyl]‐N,N‐dimethyl‐1‐propaniminium trifluoroacetate) (DOSPA) and 1,2-dioleoyl-sn-glycero -3-phosphoethanolamine (DOPE)	• Reliable in vitro transfection	• Low serum tolerance	[7, 8]
	Comirnaty (marketed, in the clinic): ((4-hydroxybutyl)azanediyl)bis(hexane-6,1-diyl)bis(2- hexyldecanoate), 2 [(polyethylene glycol)-2000]-N,N-ditetradecylacetamide, 1,2-distearoyl-sn-glycero-3-phosphocholine, and cholesterol	• Simplicity • Proven vaccination efficacy	• Side effects • Low specificity	[9, 10]
Proteins/ peptides	Mitochondrial transcription factor A fused to functional proteins in complex with DNA (TFAMoplex)	• Rational design • Biodegradability • High specificity • High serum tolerance	• Limited protection against enzymatic degradation • Immunogenicity concerns	[11, 12]
Inorganic materials	Gold nanoparticles	• Tunable and consistent size • Surface functionalization • Stability • Biocompatibility of some materials	• Inefficient endosomal escape • Limited loading capacity	[13, 14]

Those who embark on the quest to develop an optimal non-viral DNA delivery system - one that is both safe and efficient - will find themselves reliant on the precise localization and quantification of the delivered DNA at every stage of the transfection process. In this review, we critically evaluate selected fluorescence microscopy visualization methods that are applied for the detection of exogenous DNA inside cells, dissecting their advantages and disadvantages. By delving into these methodologies, we aim to equip the reader with the knowledge necessary to navigate the intricate landscape of DNA delivery, with the objective of providing tools to rationally optimize DNA carriers.

Several imaging methods can be used to track DNA inside cells [15]. Whereas fluorescence microscopy is the most common and versatile imaging technique to study the transfection process in vitro, other techniques, such as electron microscopy (EM), can also be applied for this purpose. Even though EM-based methods allow for high resolution and provide important qualitative insights, they fail to furnish quantitative information, require complex sample preparation, and do not allow imaging of living cells [16]. Therefore, they are often applied as a supplementary tracking method in combination with fluorescence-based stainings, such as correlative light and electron microscopy (CLEM) [17–19]. A review of the CLEM method was recently published by van den Dries et al. [20] and will not be discussed in this review article.

The most straightforward fluorescence-based visualization method relies on DNA covalently labeled with a fluorescent dye (“Covalently labeled DNA” section). Labeled DNA is easy to use, versatile, and allows imaging in living cells. The researcher can choose from a wide variety of fluorescent dyes with different spectral properties, quantum yields, and photostabilities [21]. A second important DNA labeling technique is the use of a DNA binding protein, e.g. the lactose repressor (lacI), fused to a fluorescent protein (FP), that is expressed by the target cell to specifically stain intracellular DNA sequences containing the appropriate DNA binding site, e.g. the lac operator (lacO) in the case of lacI (“LacO/lacI-FP” section). Lastly, labeled oligonucleotides can be used for intracellular detection of specific DNA sequences by fluorescence in situ hybridization (FISH) (“FISH” section). This technique is highly specific and sensitive as it allows for detecting and localizing low amounts of DNA molecules within a cell [22]. However, it requires extensive sample preparation for hybridization.

## The DNA delivery pathway and the challenges for DNA visualization

**Fig. 1:**
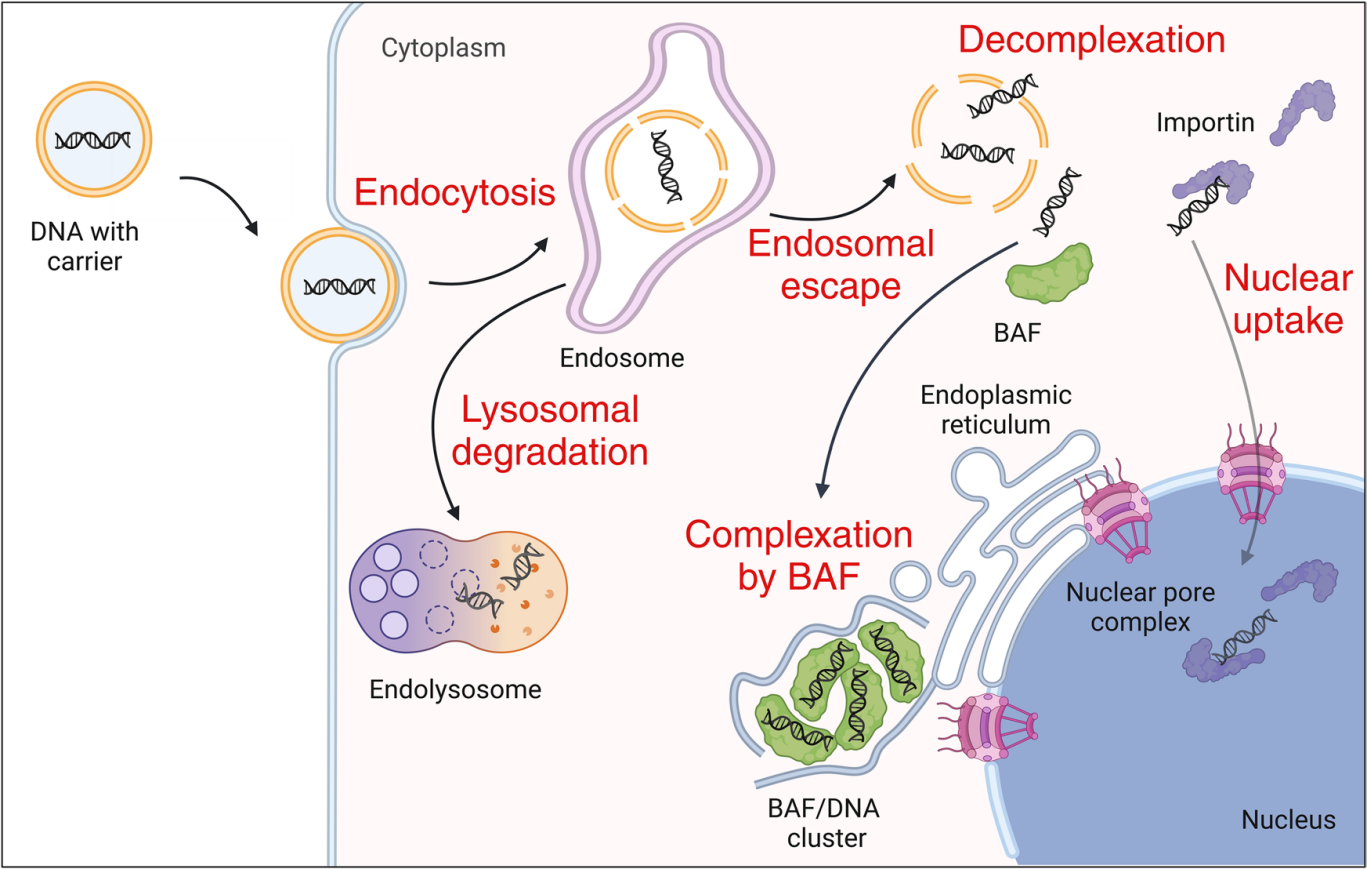
Scheme of a mammalian cell depicting the different steps of the transfection process, including endocytosis, endosomal escape, or lysosomal degradation, decomplexation after cytosolic delivery, DNA complexation by the barrier-to-autointegration factor (BAF) resulting in an endoplasmic reticulum (ER) associated BAF/DNA cluster, or direct nuclear uptake via the importin machinery through the nuclear pore complex (NPC). Created with BioRender.com

To accurately track DNA inside the cell, one has to be aware of the major hurdles in the gene delivery process and how they affect DNA detection. In this section, we introduce these obstacles from the perspective of an incoming DNA molecule (Fig. 1). The first hurdle a transfection system needs to overcome is the cellular membrane. Typically, endocytosis is the main route for cellular uptake [23–25]. When assessing the amount of DNA that is internalized by the cells, one has to consider the fact that many transfection agents bind to the cell surface via electrostatic interactions and/or hydrogen bonding [15, 26–29]. Consequently, there are two different populations of particles at the early stages of transfection – the ones associated to the cell surface and the ones in the endolysosomal system. If those are not properly distinguished, the efficiency of endocytosis can be easily overestimated. Additionally, fluorescent imaging of endosomal and lysosomal content is not trivial since the luminal conditions such as pH, salt, and lipid composition can change rapidly and may potentially interfere with the spectral properties of the fluorophore [30]. These factors must be considered in the tracking and quantification of DNA during cellular uptake.

After successful uptake via endocytosis, the nucleic acid should escape the endosomes to evade lysosomal degradation. The efficiency of this process is a critical factor for any delivery system and is yet difficult to assess with precision. The mechanisms of endosomal escape induced by the transfection agent remain a persistent matter of debate, highlighting the lack of appropriate visualization systems to study this obstacle [30–32]. In most cases, colocalization studies of endolysosomal markers with labeled DNA are performed to study endosomal escape (“Colocalization of labeled DNA with the endolysosomal system” section) [33, 34].

Following endosomal escape, it is important to track the cytoplasmic fate of the DNA [35]. Does it undergo decomplexation from the transfection agent and migrate towards the nucleus by active or passive transport? Is it recycled back to the external environment [34, 36]? Is the DNA degraded by nucleases [37–39] or negatively affected by auto phagocytic processes or innate immune responses, such as the cyclic GMP–AMP synthase (cGAS) – stimulator of interferon genes (STING) pathway [40–42]? With the help of current DNA tracking tools, it became evident that a significant fraction of cytoplasmic exogenous DNA is quickly bound and clustered by the protein barrier-to-autointegration factor (BAF), and subsequently retained in an endoplasmic reticulum (ER)-derived membrane cage [17, 43–46]. The role of these BAF/DNA clusters in the transfection process is not well understood, and they might support but concurrently also impede the nuclear uptake of exogenous DNA. Importantly, the BAF/DNA clusters form dominant structures inside the cell, which seem to contain most or all the transfected DNA [45–47]. However, with the current DNA visualization methods, the detection of weaker signals, e.g. small amounts of DNA which might have evaded BAF clustering, are masked by the very bright BAF/DNA clusters containing the bulk of the cytoplasmic DNA. One might, therefore, overlook the faint - but important - processes that could influence the outcome of the transfection. Once these processes are better characterized, which requires combining existing tracking methods with novel specific and sensitive visualization tools, they could be exploited to improve the current transfection agents.

Another important obstacle, in particular for DNA delivery, is the nuclear uptake [48]. While viruses have evolved various mechanisms to transport their DNA to the nucleus [1, 49, 50], the uptake of non-viral gene carriers through intact nuclear membranes is disputed [51, 52]. Some studies suggest that nuclear uptake of DNA mostly occurs at the end of mitosis, when the nuclear envelope (NE) reassembles and thereby incorporates exogenous DNA [19, 53–55]. Other studies argue that a fraction of cytoplasmic DNA can be actively transported through the nuclear pore complex (NPC), based on transfection experiments in non-dividing cells. This mechanism is thought to depend on transcription factors with nuclear localization signals (NLSs) that bind to specific sequences on the DNA in the cytoplasm. They then interact with the nuclear import machinery to shuttle the cargo through the NPC [56, 57]. Importantly, less than 100 DNA molecules in the nucleus can suffice to generate a measurable cellular response [58, 59]. However, since most of the transfected DNA - and thus most of the fluorescent signals - remains trapped in cytoplasmic BAF clusters, the detection of faint signals inside the nucleus remains difficult.

## Covalently labeled DNA

### Overview of DNA labeling strategies

**Fig. 2:**
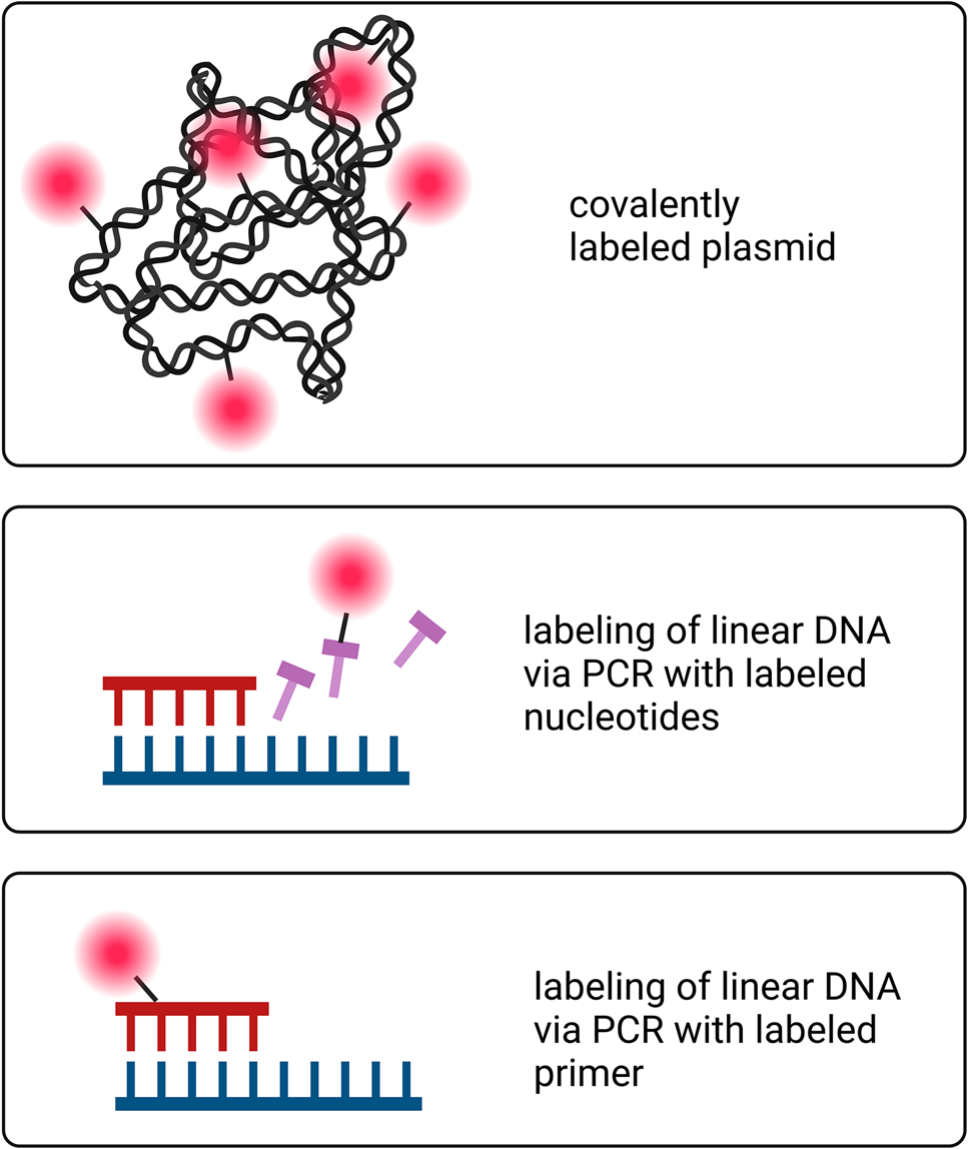
Covalent labeling of different DNA molecules. Created with BioRender.com

Fluorescently labeled DNA is the most frequent visualization method to study DNA uptake and intracellular trafficking. In a recent review, Rombouts et al. [21] discussed in detail the various dyes and labeling strategies. For a deeper insight into this topic, the reader is referred to this publication while we will focus mainly on the experimental aspects of the tracking studies. Commonly used fluorophores are rhodamine, fluorescein, and cyanines and their derivatives. These fluorophores are increasingly replaced with Alexa Fluor or ATTO dyes that offer improved brightness and photostability. Alternatively, DNA can be covalently conjugated with haptens, such as biotin. This allows to stain the biotinylated DNA by non-covalent conjugation with fluorescently labeled streptavidin, which can bind biotin even in biological fluids or inside the cell. Usually, DNA conjugation is achieved via reactive chemical groups that are incorporated into specific DNA bases (Fig. 2). Several companies offer ready-to-use labeled plasmids as well as kits for DNA labeling. The Label IT^®^ Nucleic Acid Labeling Kit form Mirus bio, for instance, consists of a Label IT^®^ linker which is coupled to a fluorophore or hapten and to a reactive group. This group reacts with (deoxy)guanine residues upon incubation with the DNA or RNA of interest [60]. Another example is the ULYSIS™ Nucleic Acid Labeling Kit from Molecular Probes [61]. With the Universal Linkage System using platinum-based chemistry, Alexa Fluor dyes are coupled to (deoxy)guanine of DNA, RNA, short oligonucleotides, or peptide nucleic acids (PNAs). PNAs can be either directly labeled or linked to quantum dots [62–64].

Besides covalent labeling via the modification of a functional DNA molecule, linear DNA can be labeled by polymerase chain reaction (PCR) (Fig. 2). This is either performed via random incorporation of fluorescently modified nucleotides in a nascent PCR product or via fluorescently labeled primers to generate linear DNA molecules with labeled termini. We will discuss the experimental benefits and limitations of these DNA labeling methods below.

#### Labeling of functional DNA molecules

With the covalent labeling of bases in an intact DNA molecule, a wide variety of DNA species can be modified, including plasmids that have been amplified in bacteria [60, 65]. This has the great advantage that the labeled end product can maintain certain properties, i.e. its degree of supercoiling or complex methylation patterns. On the other hand, the label is randomly distributed on the DNA backbone, and thus can also be linked to reporter gene sequences. Due to steric hindrance, modified bases can prevent the binding and/or activity of RNA polymerases and other DNA binding proteins. Together, these effects can negatively affect DNA transcription. This was demonstrated by Rombouts et al. [66] in lipofection experiments. The increasing number of cyanine 5 (Cy5) labels conjugated to a green fluorescent protein (GFP)-encoding plasmid first resulted in an increase in transfection efficiency in HeLa cells, followed by a strong decrease at a higher number of labels per plasmid. The authors subsequently performed nuclear microinjection with Cy5-plasmid at various labeling densities to asses the contribution of DNA transcription, independent of potential alterations on DNA uptake and intracellular trafficking. With a labeling density of 10 dye molecules per plasmid, expression decreased by 50% compared to the control plasmid. To show the accessibility of labeled DNA for enzymes such as polymerases, they carried out semiquantitative PCRs with DNA exhibiting nine different labeling densities ranging from 0 to 100 labels per plasmid. The amplification efficiency via PCR decreased starting from 25 labels per plasmid. This indicates that the modifications indeed interfered with DNA-reading enzymes.

Additionally, chemical labeling can also change the physicochemical properties of the DNA, such as its hydrophilicity or the interaction with the transfection agent [67]. To show that the association with the transfection agent (Lipofectamine or 1,2-dioleoyl-3-trimethylammonium-propane (DOTAP)/dioleoylphosphatidylethanolamine (DOPE)) was affected by the hydrophobic Cy5 label, Rombouts et al. [66] used the surfactant sodium dodecyl sulfate (SDS) at various concentrations to destabilize the transfection agent/DNA complex, and analyze the latter by gel electrophoresis. At labeling densities of up to 10 dyes per plasmid, dissociation of the plasmid DNA (pDNA) from the transfection agent/DNA complex required an SDS concentration of at least 0.01 μg/μL, whereas no dissociation was observed at 100 labels/plasmid with up to 6 μg/μL SDS. The authors concluded that at 100 labels per plasmid, the DNA was more strongly complexed by the transfection agent. In a next experiment, the release of DNA from lipoplexes was assessed in endosome-mimicking conditions by the addition of anionic lipids that are also present in the endosomal membrane. It is postulated, that mixing the lipoplex lipids with the lipids from the endosomal membrane is the driving mechanism for the release of the cargo to the cytoplasm [31, 68]. At a labeling density of 100 labels per plasmid, they observed only 20% “release” of the plasmids from the “artificial endosome” vs*.* 60% for 10 labels per plasmid and 70% for unlabeled plasmids. Taken together, these data indicate that with an increasing number of labels, the overall transfection efficiency of a plasmid can change due to effects on lipoplex properties and transcription [66]. Furthermore, the labeling degree must be carefully balanced for an optimal tradeoff between high fluorescent signal intensity and minimal impact on cellular processing.

#### PCR based DNA labeling methods

DNA labeling can also be achieved by incorporating fluorescently modified nucleotides or labeled primers in a DNA strand during a PCR reaction. The method is simple and does not require chemical conjugation steps. The use of labeled nucleotides allows to fine-tune the proportion of incorporated fluorophores by changing the percentage of modified nucleotides in the deoxyribonucleoside triphosphate mix. However, as for the chemical conjugation method described above, the labeled nucleotides may also interfere with Watson-Crick base pairing and enzyme function. In case labeled primers are used for the PCR reaction, the fluorophores are incorporated specifically at the termini of the nascent PCR product [38]. Thereby, the locus and number of modifications can be precisely controlled, which is a great advantage compared to random labeling methods. This allows the preservation of the original helix structure in the coding regions of the DNA molecule and is thus more compatible with downstream applications such as cellular expression of a reporter protein from the labeled DNA molecule. However, most DNA delivery experiments are performed with circularized DNA due to the inherent susceptibility of linear DNA to nuclease-dependent degradation in body fluids [38, 69, 70]. Thus, the linear PCR products should be circularized prior to transfection [71]. Unfortunately, the protocols for circularization often result in low yields [72].

Two additional aspects should be considered when using fluorescently labeled DNA in cellular tracking experiments. First, the probe must be sufficiently purified to ensure that it is not contaminated with free, non-DNA-associated, fluorophores. These could easily generate artefacts in cell experiments, such as bright aggregates or unnecessary background signals. Second, in case the labeled DNA is destroyed, for example by nucleases in the endolysosomal compartment [73, 74], the label might be released and continues to provide a signal. Consequently, exercising caution when interpreting tracking experiments is crucial to avoid misleading conclusions.

Besides covalent labeling, DNA can be visualized via DNA intercalating dyes [75, 76]. These dyes are almost non-fluorescent in solution but develop strong fluorescence when intercalated between DNA bases. Common examples are 4',6-diamidino-2-phenylindol (DAPI) [77] and Hoechst [78] that are both cell permeable and mainly used to stain the nuclear chromatin. Since intercalators bind DNA in a sequence-unspecific way, they cannot be used to distinguish endogenous from exogenous DNA. However, they can be applied as a co-staining technique to indicate if a labeled DNA probe, e.g. in the cytoplasm, is still intact or degraded.

### Application of labeled DNA

**Fig. 3:**
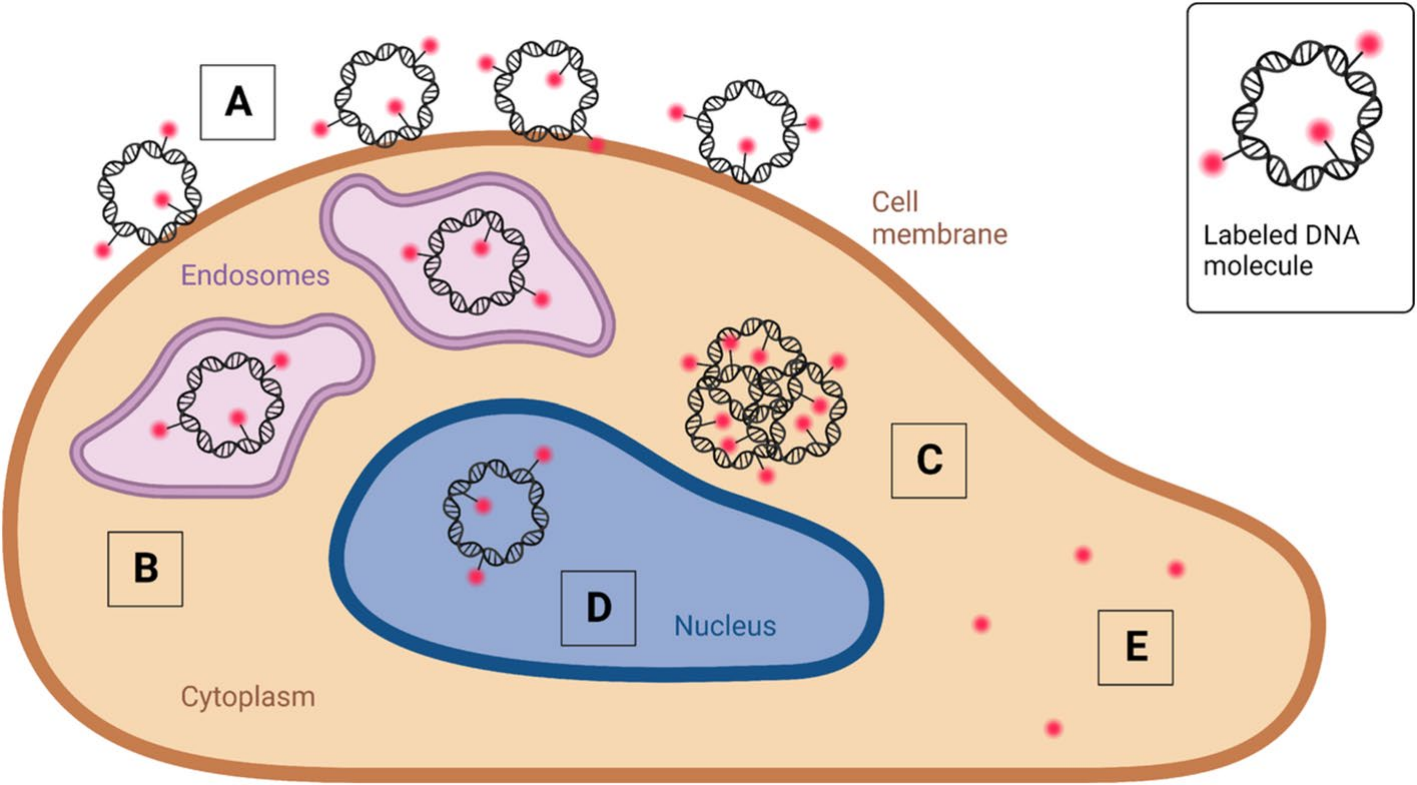
Schematic representation of labeled pDNA transfected into a mammalian cell. To focus on the localization of the DNA in the cell, DNA is displayed without its transfection agent. A large fraction of labeled DNA can stick to the cellular membrane, generating a strong extracellular background (**A**). After successful uptake, the DNA can be localized in the endolysosomal compartment (**B**). In the cytoplasm, the delivered DNA is mostly present in condensed clusters (**C**), and a small fraction of the DNA is thought to localize in the nucleus (**D**). DNA-unbound labels can create background fluorescence (**E**). Created with BioRender.com

While the use of fluorescently labeled DNA is well-established to assess the fate of the DNA during its cellular journey (Fig. 3) [34, 79–85], simple DNA labeling is not always the most suitable method and can lead to the introduction of staining artefacts and misinterpretation. Within this subsection, we examine when the use of labeled DNA is appropriate and in which situations it exhibits limitations and should be combined with additional visualization techniques.

#### Cellular uptake

The first step in the transfection process is cell surface binding and uptake. Labeled DNA is well suited to assess this step when an appropriate fluorophore is used. In our group, for instance, fluorescein isothiocyanate (FITC) labeled pDNA was applied to visually assess the cell surface binding of a protein-DNA complex on HeLa cells. The nucleus and the cellular membranes were stained with Hoechst and 1,1'-dioctadecyl-3,3,3',3'-tetramethylindocarbocyanine perchlorate (Dil), respectively [12]. Generally, labeling the cell membrane is required for the reliable localization of the transfection system outside or inside the cell. The respective images in this study revealed that most of the pDNA localized at the cellular membrane. For the control system Lipofectamine P3000 however, the extracellular signal was negligible, and most pDNA signal originated from the cytoplasmic region.

Generally, transfection agents require affinity to the cellular membrane, glycocalyx, or specific receptors to mediate endocytosis [26, 86–89]. The stronger the interactions, however, the more difficult it is to remove non-endocytosed DNA from the cellular membrane during sample preparation. In our study [12], the affinity of the DNA/protein complexes with the cell surface were so strong that it could not even be removed with harsh washing conditions involving heparin and acidic pH. In these cases, a significant amount of the labeled DNA/transfection agent complex can remain on the cell surface and might be wrongfully assigned to the endosomes or cytoplasm. This also needs to be considered when flow cytometry is used to quantify cellular uptake [82, 84, 90, 91]. Microscopy studies with co-staining of the cell membrane are required to confirm that the quantified fluorescence signal indeed originates from DNA inside the cell, as shown in the study of Liu et al. [91]. If the cell surface retention of the transfection system is very pronounced, the strong extracellular signals can complicate the tracking of the much weaker signals inside the cell. Under these circumstances, a more suitable visualization method should be used for intracellular detection of the DNA, which specifically stains the cytoplasmic DNA but not the DNA populations on the cell surface or inside the endosomes. The placO/lacI-FP system was developed for this purpose and is discussed in the “LacO/lacI-FP” section.

#### Colocalization of labeled DNA with the endolysosomal system

After successful uptake of the complexed labeled DNA, generally via endocytosis, the cargo must escape the endosome to evade lysosomal degradation. To track DNA at this stage, suitable co-stainings must be performed. Endolysosomes can be labeled in living cells with pH-sensitive fluorescent dyes such as LysoTracker™ or LysoSensor™ (Thermo Fisher). Alternatively, specific endosome-associated proteins can be overexpressed and fused to a FP to visualize a specific endosomal subpopulation. Examples are Rab5 for early endosomes, Rab7 for late endosomes, and LAMP1 for lysosomes [92]. Finally, immunostainings can be performed with antibodies that bind for instance to the early endosomal protein EEA1 [93] or the lysosomal proteins LAMP1 or LAMP2 [30, 91, 94, 95].

Several parameters must be considered when selecting an appropriate colocalization method. Immunostaining of intracellular structures, for instance, requires cell fixation and permeabilization. Cell permeabilization can lead to artefacts both regarding the endosomal localization, as well as the apparent endosomal escape of labeled DNA molecules [96–99]. Therefore, immunofluorescence studies should always be combined with other staining methods, e.g. with imaging experiments with living cells that are expressing labeled endosomal markers [33]. Vice versa, localization studies that rely on the overexpression of cellular proteins should be backed up by immunofluorescence for several reasons [100]. First, the fluorescent proteins (e.g. GFP, 24 kDa) are rather large and can impact the biological function of its fusion partner. Second, the marker proteins are often overexpressed from strong viral promoters, e.g. cytomegalovirus promoter, resulting in highly increased copy numbers with respect to the endogenous untagged protein. And third, overexpression can affect cellular processes and the abundance of the tagged protein may lead to mislocalizations and protein aggregation [100–103]. LysoTracker™ or Lysosensor™ can be applied to the cell medium in live cell experiments to stain the acidic cellular compartments due to their spectral pH-dependence. These dyes are therefore well suited for colocalization studies with transfection agents that end up in the maturating endolysosomal system [104]. Majzoub et al. [34] used LysoTracker™ Red, Rab11-GFP labeled recycling endosomes, and Cy5 labeled plasmids to study the endosomal pathways of cationic lipid nanoparticles (NPs). By co-staining the endosomes with LysoTracker™ Red and with various Rab-GFP proteins (Rab5, 7, 11, and 9), the authors found that Lysotracker™ significantly colocalized with Rab7 and Rab9, confirming that this dye stained the endolysosomal pathway. Little colocalization was detected between LysoTracker™ and Rab5 or Rab11, most likely because Rab5 is a marker for the non-acidic early endosomes, and Rab11 for the non-acidic recycling endosomes. With this finding, they confirmed that a staining of Rab11 for the recycling pathway, and the application of LysoTracker™ to identify the degradative endoslysosomal pathway was enough to track the NPs along the endosomal route. In another set of experiments, they incorporated a mixture of Cy5 labeled and unlabeled plasmids into different NPs, that were subsequently incubated with Rab11-GFP expressing prostate cancer cells. The cells were also stained with LysoTracker™ Red and Hoechst. Colocalization of the three fluorescent signals (Cy5-pDNA, LysoTracker™ Red, Rab11-GFP) revealed that the NPs ended up in both, the recycling and the degradative endolysosomal pathway. With this co-staining method, it was found that the recycling pathway was prevalent for NPs with high membrane charge densities (0.021 e/Å^2^), whereas the degradative pathway dominated at low (0.0061 e/Å^2^) and very high membrane charge densities (0.025 e/Å^2^).

#### Impact of fluorophore properties on visualization of endosomal escape

Generally, data obtained with acidic organelle markers such as LysoTracker™ should be analyzed with caution in the presence of substances that affect the acidification of the endosomal lumen, such as transfection agents with alleged buffering capacity (e.g. poly(ethyleneimine) (PEI)) or small chemicals (e.g. bafilomycin or chloroquine) as the changes in luminal pH may affect the labeling [32, 80, 84, 90, 105]. In these cases, methods involving the overexpression or immunostaining of endolysosomal proteins should be preferred. Figueroa et al. [84] for instance, used Cy5 labeled pDNA bearing a GFP reporter gene for transfection with gold–poly(amidoamine) or PEI in SK-BR3 or CT26 cells. With LysoTracker™ Yellow, they assessed the endolysosomal localization of the delivered DNA. In the SK-BR3 cells, after transfection, the yellow fluorescence signal was weak, although several cells showed GFP reporter gene expression after 24 and 48 h. This could indicate that the SK-BR3 cells did indeed take up the DNA, but endosomal acidification was possibly affected by the transfection agents. Without the presence of acidic pH, LysoTracker™ Yellow signal might be reduced. Thus, to study uptake and endolysosomal localization, the use of co-stainings with proteins resident at the respective cellular compartment would have been necessary to validate the subcellular Cy5 localization.

Next, the selection of the DNA labels should be based on the nature of the experiment and the environment of the assessed cellular compartment. Fluorescent dyes are often not pH stable, their signal intensity can vary in different microenvironments and be quenched (e.g. at high concentrations in a NP). FITC, for instance, is pH sensitive, and is only weakly fluorescent below a pH of 5 [106]. Thus, its application for tracking the endolysosomal pathway is not optimal [107, 108]. Nevertheless, FITC is often used to track DNA, also in endolysosomes [31, 85]. Rehman et al. [31] for instance, used FITC labeled oligonucleotides-PEI complexes to study the proton sponge effect. The study reported a low FITC signal in the endosomes, which was explained by the fluorescence quenching in the complexes. Upon endosomal escape, a sudden burst of FITC signal was detected, which was attributed to PEI/DNA decomplexation in the cytoplasm. An alternative explanation would be the quenching of the FITC signal inside the acidic endolysosomes, followed by dequenching upon release into the neutral cytoplasm [108]. To confirm each one of these hypotheses, a pH-insensitive dye, e.g. Alexa dye, should have been used as a control [107].

#### Stimuli-responsive systems to detect endosomal escape

Ideally, a visualization system for endosomal escape would only emit a signal upon leaving the endosome. Such a system could be inspired from stimuli-responsive drug delivery systems that respond to changes in redox or pH conditions [30, 109, 110]. To study the assembly and drug release by cleavage of a responsive linker, fluorophores with aggregation-induced emission (AIE) are commonly employed [111–113]. Sun et al. [114] for instance, constructed a redox-sensitive drug delivery system with AIE. They conjugated the AIE fluorophore to poly(ethylene glycol) (PEG) via disulfide bonds. Upon simulating the reducing conditions of the cytoplasm, the system disassembled, resulting in a loss in the fluorescence signal. Such an approach could be adapted to DNA tracking assays. Another strategy to quantify endosomal escape was recently developed to detect proteins entering the cytoplasm by Teo et al. [115, 116]. The technique is based on a complementary split luminescent marker protein and requires a stable cell line expressing one part of the split protein. The other split partner is coupled to the delivered cargo. Once the cargo entered the cytosol, the split partners mature to form an active bioluminescent enzyme. While this technique is well suited for protein tracking, the coupling of proteins or peptides to DNA holds difficulties and may affect the structure and function of a DNA molecule. It could, therefore, alter the cellular fate of the DNA if not properly optimized for this application.

#### Nuclear uptake

Following successful cytosolic delivery, the DNA needs to travel across the cytoplasm, and eventually enter the nucleus where it is transcribed [117]. Independent of the applied transfection method, DNA forms large dense clusters in the cytoplasm, in a process that is dependent on BAF and its interaction partners. When fluorescently labeled DNA is used, these clusters, which are often located close to the nucleus, become very bright and might contain thousands of DNA molecules [118, 119]. This is problematic from a gene delivery point of view but also makes the detection of single, soluble exogenous DNA molecules in the nucleus difficult.

**Fig. 4:**
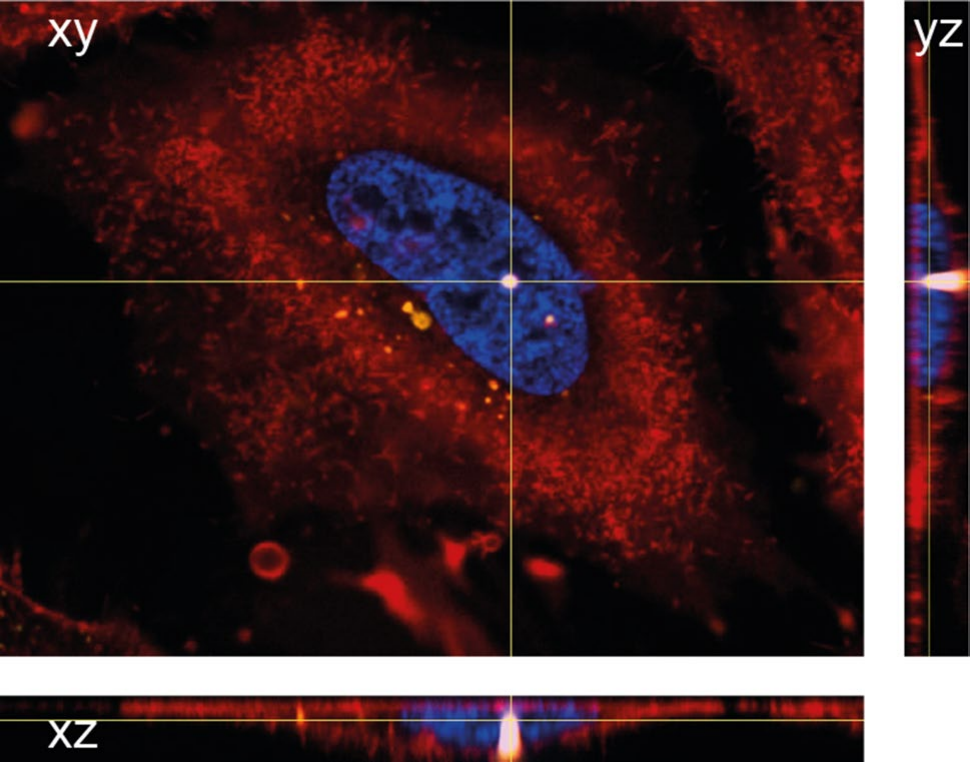
HeLa cell transfected with 1 µg of Cy3 labeled pDNA with X-tremeGENE™ 9 DNA Transfection Reagent 20 h before imaging via confocal spinning disk microscopy. Z-stack slice size: 0.2 µm, 27 stacks. Scale bar: 10 µm. Nucleus stained with Hoechst 3342 (blue), Cy3-pDNA (yellow), plasma membrane stained with CellMask™ DeepRed (red). Detailed methods in Supplementary information

The image shown in Fig. 4 is representative for many studies using labeled DNA to detect nuclear localization [79–81, 91, 118, 120]. The imaging settings were adjusted to also see weaker DNA signals. This resulted in the overexposure of very bright DNA clusters. Note that the cluster shines through almost the entire z-stack and could, therefore, be assigned to any compartment of the cell, including the cell surface. Kamiya et al. [81] for instance, described bright DNA clusters in one of the first studies about the intracellular trafficking of exogenous DNA delivered by cationic liposomes. NIH3T3 cells were transfected with rhodamine labeled plasmids complexed with Lipofectamine PLUS, fixed at several timepoints after transfection, and imaged. SYTO24 was applied as counterstaining for genomic and uncomplexed DNA, and colocalization with the rhodamine-pDNA was performed. The authors detected colocalization of the rhodamine signal with the nuclear staining as early as 30 min after transfection. Interestingly, in their images, the clustered signals, deriving from the overlay of the SYTO24 and rhodamine signals, were also partially overexposed as could be seen from blurry cluster borders and missing substructures inside the cluster.

Even though many studies assert to detect the signal of labeled DNA inside the nucleus with a simple co-staining of the nuclear chromatin, we believe that the correct identification of exogenous DNA inside the nucleus requires additional evidence. The labeled DNA clusters might also be enclosed in NE-like membranes in close vicinity to the nucleus [47], and/or entrapped in nuclear invaginations [121, 122]. These membranous invaginations of the NE are channels with a diameter of 100–1000 nm that span the nucleus, and are common in cancer cells [123, 124]. Ferri et al. [125] found that some Cy3-pDNA signals that seem to be localized inside the nucleus partially overlap with a NE-membrane staining. They transfected CHO-K1 cells with Cy3-pDNA containing lipoplexes and performed confocal laser scanning microscopy 3–6 h after transfection with Hoechst stained nuclei. To visualize the NE, the amphiphilic membrane dye FM4-64 was employed. It was found that all Cy3-pDNA clusters in the nuclear region were associated with the NE staining, suggesting that these clusters were, at least partially, entrapped in nuclear invaginations. Interestingly, Ondrej et al. [126] observed that fluorescently labeled DNA signals, which seemed to be situated inside the nucleus were colocalizing with actin fibers and lamins. The authors concluded that the nucleus must therefore contain an actin cytoskeleton, which, to our knowledge, has never been reported. We hypothesize that the observed DNA was not inside the nucleus, but rather in actin-containing nuclear invaginations [127, 128]. These findings underline the importance of inner nuclear membrane co-stainings for the nuclear localization of transfected DNA because solely staining the chromatin is imprecise and can be misleading. Such co-stainings could be achieved either by NE-dyes or by visualizing inner nuclear membrane proteins, such as emerin, with both, immunofluorescence and overexpression of the respective FP fusion proteins. Introducing an additional technique like immuno-CLEM could further confirm an apparent nuclear presence of the delivered genes as it allows a resolution below 10 nm and the visualization of membranous structures.

## LacO/lacI-FP

### Principle

The second DNA tracking method that is discussed in this review allows to visualize DNA inside cells with very little extracellular background and is based on the cellular expression of DNA binding proteins fused to a FP. The fluorescent DNA binder is produced by the target cell and, therefore, mostly found within the cytoplasm, and the nucleus. Once DNA with the specific targeting sequence is delivered into the cell, the fusion protein strongly localizes at these sites, effectively staining the DNA molecules. Theoretically, any DNA binding protein could be used for this purpose, but most systems are based on gene regulators with very high affinity and specificity for short genetic sequences, such as the bacterial lactose repressor lacI [129, 130], or tetracycline repressor [131, 132].

The advantage of these tracking systems is their compatibility with living cells for the specific detection of intracellular DNA. DNA in the extracellular space, i.e. on the plasma membrane or in the endosomes, is almost invisible since the proteins are found in the cytoplasm and are barely secreted. However, the fusion proteins are produced by the cell in excess to the delivered DNA. Consequently, the unbound protein always creates some fluorescence background inside the cell, which complicates the detection of faint DNA signals, i.e. deriving from single DNA molecules. The method is thus mainly applied to visualize large cytoplasmic DNA aggregates [19, 47, 119, 133].

**Fig. 5:**
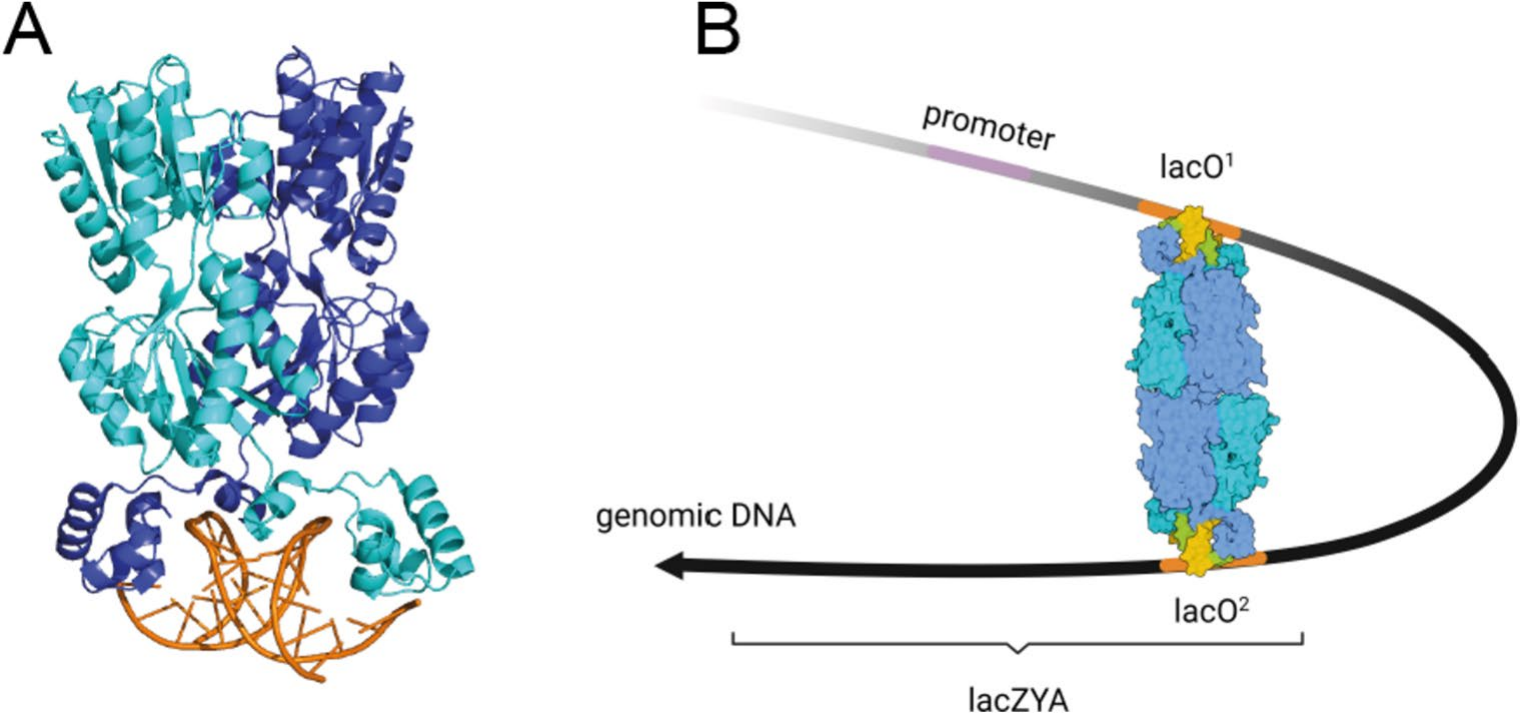
**A** Structure of the dimeric lac repressor/operator O1/ONPF complex. The dimer is displayed bound to the lacO sequence. Light and dark blue: lacI dimer, orange: lacO DNA strands. PDB code 1JWL [147]. **B** Tetrameric LacI simultaneously binds to two operator sites, leading to DNA looping. Created with BioRender.com

Here, we primarily describe the well-characterized lac operator/repressor (lacO/lacI) system. It is derived from the bacterial gene repressor lacI, which binds its operator site, lacO, sequence-specifically with an in vitro dissociation constant (K_D_) of 10 pM [134, 135]. The lacO sequence is 24 base pairs long (5′ - TGGAATTGTGAGCGGATAACAATT - 3′) [136]. As depicted in Fig. 5, wild-type lacI is a homotetramer that binds two operator sequences that are apart from each other on a DNA strand, thus bending it and serving as repressor of gene expression [137–140]. In the presence of lactose, lacI undergoes a conformational change and releases the DNA which allows the transcription of the genes LacZ, LacY, and LacA of the lactose metabolism [141–143]. To evade DNA looping, a mutant of lacI was generated, which is not able to form the homotetramer [144–146].

The application of the lacO/lacI system for the intracellular and sequence-specific detection of DNA has two requirements. First, cells are needed that (over)express lacI-FP. Ideally, a stable cell line is produced to obtain homogenous lacI-FP expression in the cell population. Second, a DNA construct bearing a lacO sequence is required. To increase the signal-to noise ratio of lacI-FP bound to lacO vs*.* unbound lacI-FP inside the cells, plasmids with multiple lacO repeats were created. In 1996, a DNA construct with 256 operator repeats was designed by Robinett and Straight et al. [129, 130] to visualize chromosome dynamics in yeast. This DNA construct was produced via consecutive cloning cycles, using an 8-mer of direct lacO repeats, developed by Sasmor and Betz in 1990 [148] that was inserted in a pUC18 derivate vector. Due to recombination, the plasmid became increasingly unstable with increasing number of repeats. Today, plasmids with the 256 lacO repeats (placO) are available. These plasmids are rather large, with sizes ranging from 12–15 kbp. This should be considered when performing transfection experiments, since standard expression plasmids are often about 5 kbp in size. The length of a DNA molecule affects the intracellular mobility and transfection efficiency [149], indicating that the plasmid size influences its nuclear uptake. Interestingly, Wang et al. [150] found, that only 13 lacI proteins are bound to a sequence of 256 tandem lac operators. This finding suggests that the lacI proteins cannot bind to lacO sequences close to each other due to a local distortion of the DNA helix. Furthermore, it cannot be ascertained that the lacO plasmids still carry 256 functional repeats of the lacO sequence, as the intactness of the repeats is difficult to assess via sequencing or PCR [151].

### Application

**Fig. 6:**
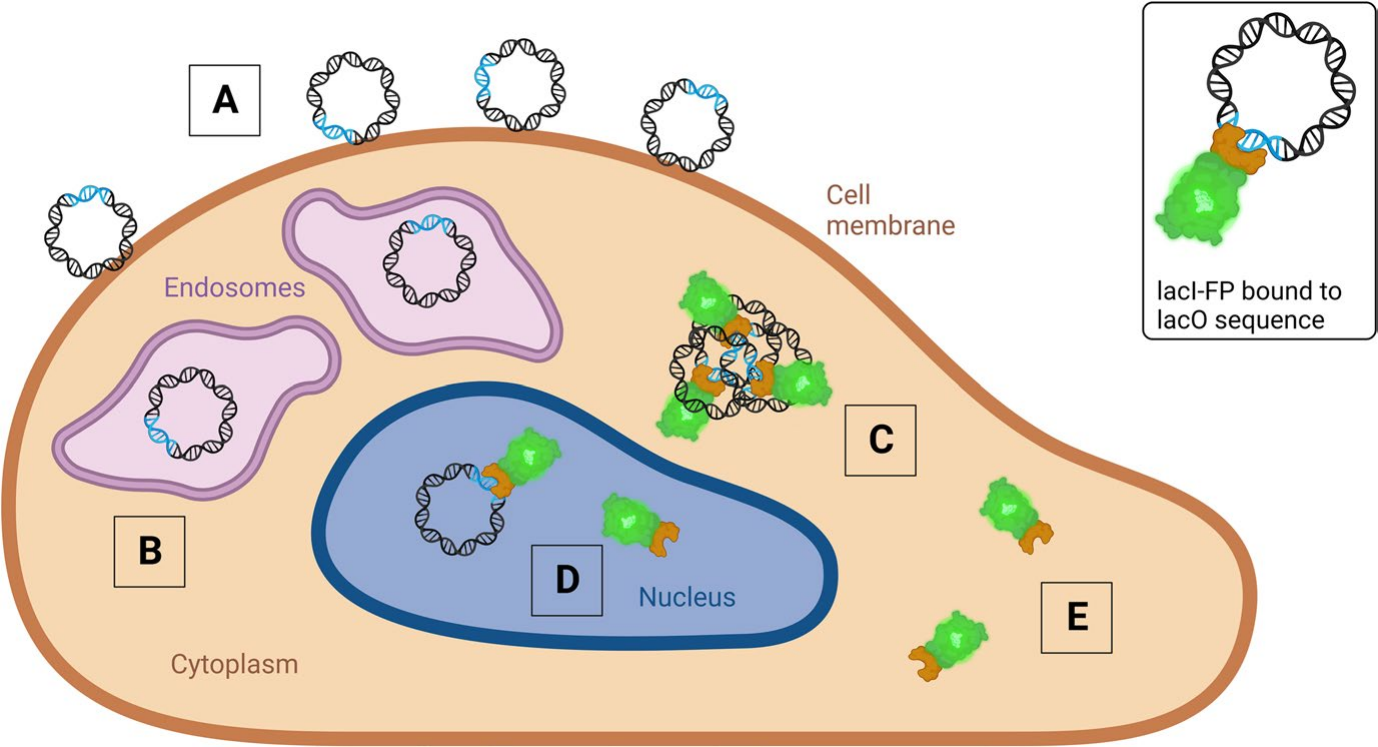
Schematic representation of placO transfected to a mammalian cell expressing lacI-FP. A large fraction of placO might stick to the cellular membrane (**A**) which is, however, not bound to lacI and thus not visible. The same applies for placO inside the endosomes (**B**). In the cytoplasm, dense placO clusters are formed which are visible as bright lacI-FP puncta (**C**). Some lacO plasmids might have entered the nucleus, however, they are difficult to detect due to signal from lacI-FP that eventually binds genomic DNA unspecifically (**D**). Background from unbound lacI-FP is also present in the cytoplasm (**E**). Created with BioRender.com

The placO/lacI-FP system has often been applied to track the fate of various DNA sequences inside the cell. This includes the visualization of transfected DNA (Fig. 6) [19, 47, 152], but also lacO sequences artificially inserted into the genome, e.g. for the examination of chromatin dynamics [129, 130], the assessment of genetic loci [145], characterization of bacterial chromosome arrangements [153], and for studying transcription dynamics in mammalian cells [154]. The first lacO tracking experiments were performed after viral transduction. Kanda et al. [152] used the 256 lacO repeats [129], incorporated in the Eppstein Barr viral vector (EBV) and delivered it to COLO320DM cells via electroporation. After expanding the cell line, the cells were transduced with another EBV vector bearing the lacI-FP gene to show where the EBV-lacO had integrated. These integration sites were then visible as punctate staining in the nucleus and the results were confirmed by FISH.

Shimizu et al. [133] demonstrated the feasibility of the lacO/lacI-FP DNA staining system with microinjection experiments in HeLa cells. They developed a HeLa cell line via transduction with the EBV vector bearing the lacI-FP gene [152]. To measure the intracellular DNA mobility, the cytoplasm or the nucleus were microinjected with various DNA constructs containing 32 lacO repeats either as a short, linearized molecule with only 325 bp, or as a long, linearized molecule with 15,080 bp, or as a 15,080 bp plasmid. LacI-FP bound the DNA directly after injection. Small linear DNA was found to be highly mobile in the nucleus, whereas larger DNA appeared to form immobile aggregates. Interestingly, the DNA signal quickly faded in the nucleus after nuclear injection and seemed to relocate to the cytoplasm, which the authors attributed to an underlying nuclear export mechanism. Similar findings were described using additional methods such as FITC labeled DNA and FISH.

**Fig. 7:**
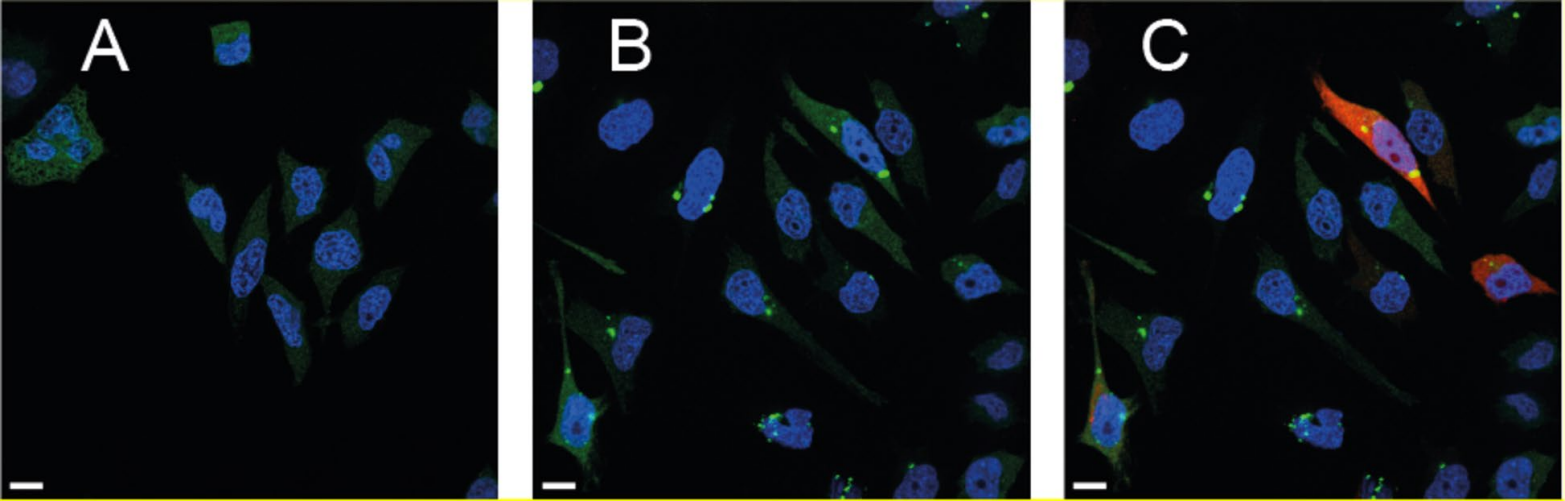
lacI-GFP HeLa cells were transfected with a plasmid with 265 lacO repeats and a gene for a red fluorescent protein (RFP) (placO-RFP) with X-tremeGENE™ 9 Transfection Reagent. 0.5 μg DNA was applied for 50′000 cells. Cells were fixed with 4% PFA one day after transfection. Nuclei were stained with Hoechst and imaged with a confocal spinning disk microscope. Detailed methods are provided in the Supplementary information. Untreated lacI-GFP HeLa cells (**A**); Cells transfected with placO-RFP (**B**, **C**). Displayed channels: **A** GFP; **B** GFP, Hoechst; **C** GFP, Hoechst, RFP. Scale bar: 10 μm. The HeLa-lacI-GFP cells were a kind gift from Professor Tokuko Haraguchi, Osaka, Japan

More recently, the lacO/lacI-FP-based tracking system was used in several studies to analyze the cellular fate of exogenous DNA after non-viral gene delivery [19, 47, 119]. Bright lacI-FP clusters were consistently observed in the cytoplasmic region of the cell, independent from the used transfection system. Figure 7 shows the typical intracellular lacI-FP clusters in transfected cells, but also the background of unbound lacI-FP that impedes the detection of faint signals.

Wang et al. [119] applied the lacO/lacI-FP system to study the partitioning of exogenous DNA clusters during mitosis. They created a stable cell line via viral transduction expressing lacI-GFP or lacI-mCherry with a NLS (lacI-FP-NLS) to minimize the cytoplasmic background of unbound lacI-FP and with a mutation of lacI at amino acid 1–358 to evade tetramerization [145]. HeLa and Madin-Darby canine kidney cells stably expressing lacI-GFP-NLS or lacI-mCherry-NLS were transfected with placO and 24 h later, the living cells were observed by confocal microscopy. The authors described the occurrence of bright lacI-GFP-NLS foci in the cytoplasm. With immunostaining of GFP, and of the placO sequence, they confirmed that the lacI-GFP-NLS foci colocalized with the placO sequences. Further, they performed co-stainings, such as the ER membrane protein Sec61 and found that the placO clusters were associated with membranes that shared features of both, the inner nuclear membrane, and ER membranes. In our opinion, lacI-FP tracking systems containing a NLS has two limitations. First, the NLS results in an increased FP background signal in the nuclear region, making the tracking of weak DNA signals inside the nucleus even more difficult [35, 59, 117]. Thus, to detect nuclear localization of DNA, the authors performed an additional tracking experiment with rhodamine labeled DNA and subsequent lamin-B1 immunostaining to visualize the nuclear rim. According to the authors, this allowed the detection of faint nuclear plasmid signals. The second issue is the possible effect of the NLS signal on the cytoplasmic movement of the transfected DNA. The lacI-FP-NLS molecules bind the transfected DNA with high affinity. Simultaneous interactions of the NLS with the nuclear import machinery, for example, could artificially affect the mobility of the DNA, for example, its nuclear import, and should therefore be applied with caution for DNA tracking [35, 57, 155].

In 2023, the membrane structure that surrounds the cytoplasmic DNA clusters was characterized in detail [47]. Methods similar to those described above were applied, but time-lapse imaging of the HeLa cells expressing lacI-FP-NLS cells was performed immediately after transfection to observe placO/lacI-FP-NLS cluster formation. The possibility to analyze living cells at early time points is a major advantage of the lacO/lacI-FP system, since extracellular DNA produces very low fluorescence background. Haraguchi et al. [53] have also applied the lacI-FP-system (without NLS) to track cytoplasmic DNA clusters, particularly during mitosis. In this study however, a plasmid with 265 lacO repeats also encoded RFP to track the timing of reporter gene expression, which made the plasmid even larger (about 15 kbp). The authors performed co-stainings of the lacO/lacI-GFP clusters with the DNA clustering protein BAF and the transmembrane protein emerin via immunostaining, and tracked the fate of the clusters during mitosis. They observed that the placO/lacI-GFP cluster dispersed into smaller puncta at the beginning of mitosis, and that some of the fragmented clusters were incorporated into the reforming nucleus in telophase. The authors further assessed the kinetics of RFP expression and found that it mostly started after mitosis. Together these findings suggest, that – at least for very large plasmids – the major uptake route into the nucleus occurs during nuclear breakdown and not through the NPC in interphase. For DNA visualization, the authors did not only rely on lacI-GFP fluorescence but also immunostained the lacI-GFP with an anti-GFP antibody and with CLEM and immuno-CLEM to detect the NE and ER membranes in the reforming nucleus.

The lacO/lacI-FP system allows to specifically visualize DNA inside the cell, especially in combination with other tracking techniques. However, the question remains whether it mainly reveals large cytoplasmic accumulations of DNA, while weaker - and maybe relevant - signals are overlooked (Fig. 6). It might be beneficial to invest in the development of a more efficient and versatile system that would not require a DNA sequence with hundreds of repeats, and - preferentially - would not require a stable reporter cell line.

## FISH

**Fig. 8:**
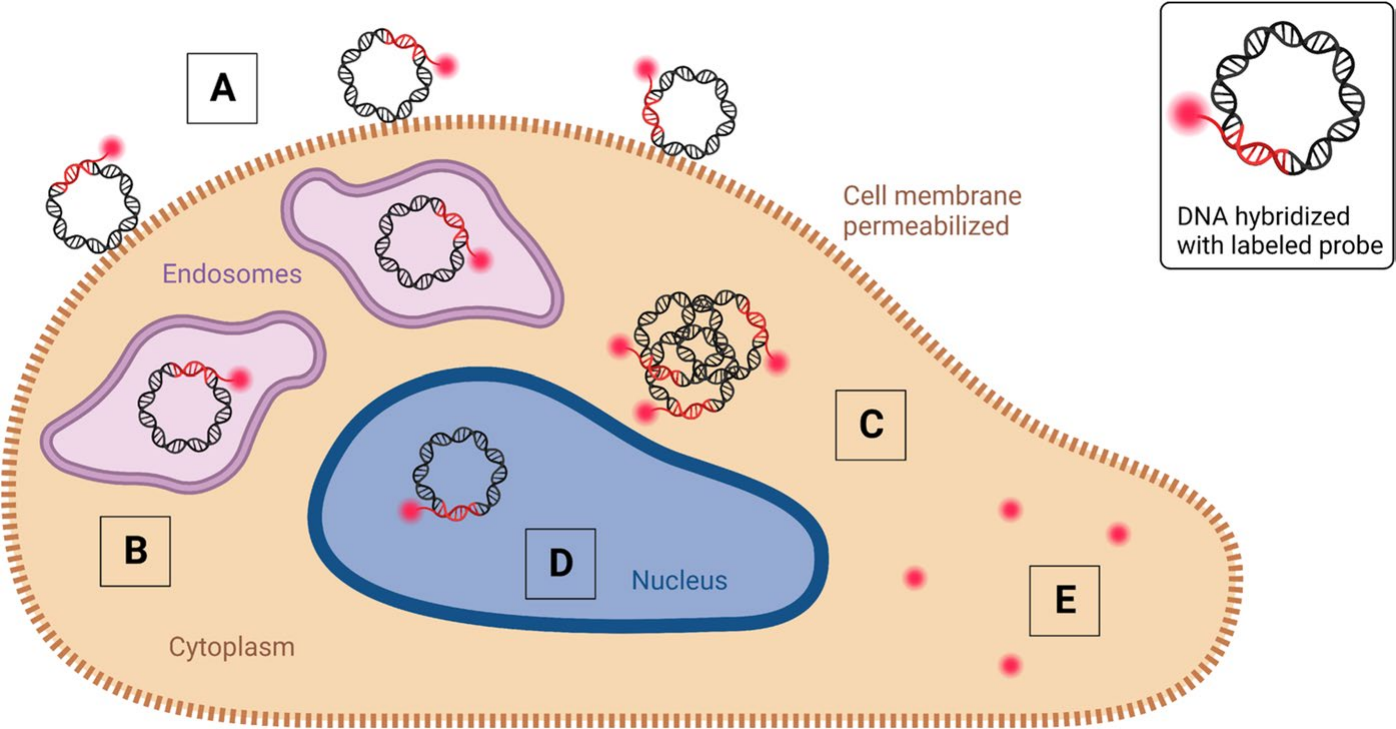
Schematic representation of plasmids transfected to a mammalian cell and subsequent visualization via FISH. Due to cell permeabilization, extracellular background from DNA hybridized with a specific labeled probe might be weak (**A**). DNA visualized in the endoslysosomal compartment (**B**). In the cytoplasm, the delivered DNA is mostly present in condense clusters (**C**), a small fraction of the DNA is thought to localize in the nucleus (**D**). Labels that detached can promote background fluorescence (**E**). Created with BioRender.com

FISH enables to detect specific DNA or RNA sequences within fixed and permeabilized cells. It employs labeled antisense oligonucleotides that are complementary to a known target DNA or RNA sequence. Hybridization follows Watson-Crick base pairing [156], with a typical probe length between 10 and 25 nucleotides. The concept for in situ hybridization was first described by Gall and Pardue in 1969 [157, 158], who employed radiolabeled probes. The use of fluorescence as a safer alternative compared to radiolabeled nucleotides was implemented by Rudkin and Stollar in 1977 to detect RNA-DNA hybrids in Drosophila melanogaster [159]. Following further developments of the FISH method, Pinkel et al. [160, 161] applied it to detect trisomy 21. Later, FISH played a pivotal role in the human genome project [162, 163], where it was exploited to observe specific gene locations on chromosomes, as well as in numerous applications in genetics and molecular biology [164–166]. FISH is very well suited for genome mapping, but it is also used to track delivered DNA inside cells (Fig. 8) [37, 47, 155, 167].

The sample preparation for FISH includes the fixation and permeabilization of the cells to make the target DNA accessible for the probes. Also, the DNA must be denatured to allow hybridization of the oligo-DNA duplex. This is usually achieved by heating and the addition of chemicals like formamide [21, 168]. Between the steps, the sample is washed extensively to remove the applied chemicals and the unbound probes. Protocols for sample preparation are found in several publications [47, 169–172].

FISH probes mainly comprise labeled oligonucleotides that can be designed and purchased from various suppliers (Microsynth, Jena Bioscience). The probes can also be produced via the “nick translation” method, in which a plasmid is enzymatically nicked, followed by a polymerization and ligation step to fill the single strand breaks using a mix of unlabeled and labeled nucleotides [167, 171, 173]. This method provides a random set of labeled probes that bind to the DNA template. Probes can also be labeled indirectly, e.g. via biotinylation, and subsequent conjugation to labeled avidin. In addition, immunohistochemical targets can be conjugated to the FISH probe for antibody-based labeling strategies, referred to as Immuno-FISH. One example of this is the incorporation of digoxigenin (DIG)-dUTP into the FISH probe, followed by the in situ staining of the probe with fluorescently labeled anti-DIG-antibodies [37, 167].

One of the first studies that applied FISH to trace delivered DNA inside the cell was conducted in 1997 by Dean [155]. DNA was microinjected into the cytoplasm of various mammalian cell lines and the impact of the strong simian virus (SV)40 promoter on nuclear import was assessed. The resulting images showed FISH signal evidently inside the DAPI counterstained nucleus after 8 h when DNA bearing the full-length SV40 cassette was microinjected, whereas bacterial DNA sequences and DNA containing only parts of the SV40 sequence did not localize in the nucleus. The author concluded that the SV40 sequence was required to promote nuclear uptake of the injected DNA. However, also in this method, a counterstaining of the nuclear membranes would be necessary to reliably conclude that DNA molecules were indeed transported from the cytoplasm to the nucleus.

The main issue when applying FISH for localization of the DNA inside cells is the harsh sample preparation protocol. It is generally accepted, that fixation and permeabilization of specimens are prone to induce artefacts [97, 174, 175]. In particular, this is problematic for the exact localization of single DNA molecules inside the rather mobile endolysosomes or in the cytoplasm [98]. During treatment with the respective chemicals and the many wash steps, small cellular compartments might dislocate or break, interfering with correct signal assignment [99]. Hence, many gene delivery studies include FISH only as an additional visualization tool, particularly when sequence-specificity is required [152, 176]. Wang et al. [119] for example, used FISH to confirm that cytoplasmic lacI-FP clusters contain the delivered plasmid. Therefore, the presence of placO was demonstrated both in living cells by lacI-FP staining as well as with the FISH method. In another study performed by this group, FISH probes were employed to show that cytoplasmic DNA clusters formed after DNA delivery also contain telomeric DNA [47]. However, in some cases the harsh manipulation for FISH staining prevents the combination with co-stainings. Schenkel et al. [47] for example, could not use an immunostaining of the endogenous protein emerin as the antibody did not survive the FISH protocol.

Taken together, FISH is a useful tool to stain DNA sequences with high sequence specificity and a minimally low limit of detection. For the detection of specific gene loci inside the nucleus, this method can be used. Large cellular structures, such as chromatin can be preserved more easily by fixation and permeabilization and are, therefore, less susceptible to introducing artefacts. Comparably small plasmids, on the other hand, might be translocated or removed during the harsh sample preparation [99], which should be considered in FISH-based DNA visualization studies. Generally, FISH should always be used in combination with other DNA visualization techniques that require minimal sample manipulation, ideally live cell imaging.

## Conclusions and outlook

**Fig. 9:**
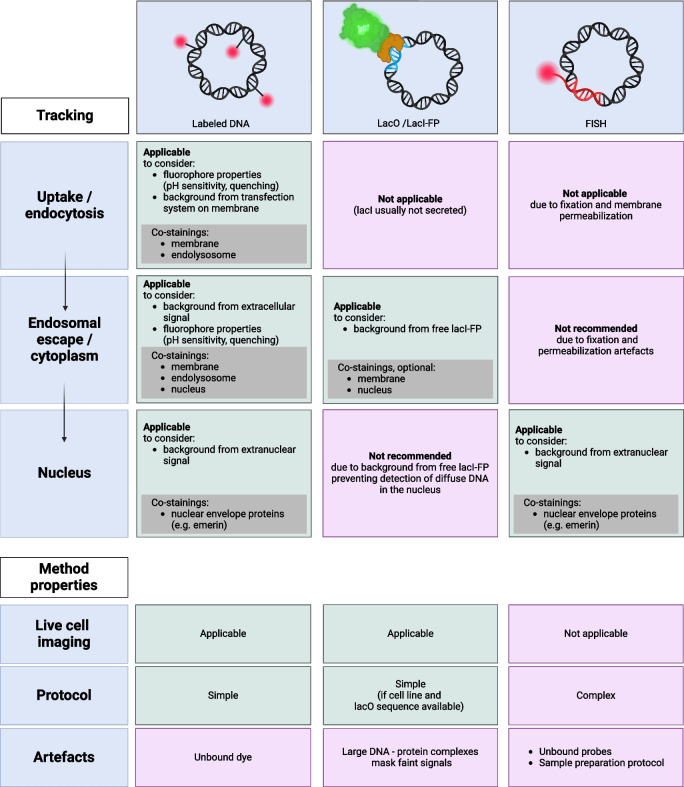
Overview for planning a DNA tracking study. Created with BioRender.com

Tracking DNA molecules inside the cell remains challenging even though many labeling techniques are available. These techniques, summarized in Fig. 9, should not be applied individually, but rather in combination, to reliably visualize and quantify the delivered DNA at the various stages of the transfection process. Labeled DNA is well suited to detect DNA on the cell surface, as well as in the endolysosomal system and in the cytoplasm. However, appropriate co-stainings should be performed to accurately assign the signals to any of those compartments. DNA binding proteins are an elegant tool to stain DNA specifically inside the cells. The often used lacO/lacI-FP system, however, should be developed further, for example by using other DNA-binding proteins that allow to track DNA in a sequence independent manner, e.g. BAF [177]. With FISH, DNA can be readily detected with high sensitivity, but the method is limited to fixed cell imaging and should not be applied to quantify uptake and endosomal escape.

The largest limitation of all the currently available DNA tracking methods is the difficulty to reliably detect DNA inside the nucleus. This is problematic since nuclear uptake is a crucial step for efficient DNA delivery and thus important for the development of any non-viral DNA transfection agent. For intranuclear localization, we recommend the use of labeled DNA or FISH but only in combination with the co-staining of the inner nuclear membrane. The colocalization with a chromatin staining, such as DAPI, is imprecise and can result in misleading conclusions about the efficiency of nuclear uptake. The development of nuclear transfection visualization tools is a great challenge because the nuclear lumen and the cytoplasm have very similar biochemical properties, e.g. pH and ion composition, and all nuclear proteins are produced in the cytoplasm. Some processes, including DNA transcription/RNA production, are, however, exclusively found in the nucleus and could be exploited for novel DNA detection techniques. As the delivered genes might be present in low copies inside the nucleus, single molecule imaging techniques, such as DNA-PAINT [178], should be further developed.

To analyze and subsequently manipulate the interaction of non-viral transfection agents with cellular structures, the creation of innovative DNA tracking techniques is pivotal alongside the careful combination of existing tools.

### Supplementary Information

Below is the link to the electronic supplementary material.

Supplementary file1 (DOCX 17 KB)

## Data Availability

The datasets generated during the current study are available from the corresponding author on reasonable request.
