# Supplementary material for: The intracellular visualization of exogenous DNA in fluorescence microscopy
Source: Drug Deliv Transl Res. 2024 Mar 25;14(8):2242–61. doi: 10.1007/s13346-024-01563-4 (PMC11208204; doi:10.1007/s13346-024-01563-4)
Supplement: Supplementary file 1 — Supplementary file1 (DOCX 17 KB) [file 13346_2024_1563_MOESM1_ESM.docx]

# Supplementary Information

for

The intracellular visualization of exogenous DNA in fluorescence microscopy

Review in Drug Delivery and Translational Research

**Christina Greitens^1^, Jean-Christophe Leroux^1^ *, Michael Burger^1^ ***

^1^Institute of Pharmaceutical Sciences, Department of Chemistry and Applied Biosciences, ETH Zurich, 8093 Zurich, Switzerland

*Corresponding authors

[Michael.Burger@pharma.ethz.ch](mailto:Michael.Burger@pharma.ethz.ch)

[jleroux@ethz.ch](mailto:jleroux@ethz.ch)

## Material and Methods

**Consumables**

Dulbeco’s Modified Eagle Medium (DMEM) high glucose GlutaMAX, fetal bovine serum (FBS), penicillin-streptomycin, 0.25% trypsin-EDTA, phosphate buffered saline (PBS), OptiMEM^®^ Reduced Serum Medium, Live Cell Imaging Solution, and CellMask™ Deep Red Plasma Membrane Stain were obtained from Thermo Fisher Scientific. Triton X‐100, Paraformaldehyde, Hoechst 33342, and Mowiol 4‐88 were obtained from Sigma Aldrich Chemie GmbH. X-tremeGENE^TM^ 9 DNA Transfection Reagent was obtained from Roche. G418 disulfate solution was obtained from AppliChem. μ-slide 8 well chambered coverslips glass bottom were obtained from ibidi. Cover glasses were obtained from Marienfeld. Glass specimen were obtained from Menzel-Gläser.

**Cell Culture**

HeLa cells (ATCC CCL‐2) were obtained from ATCC and HeLa lacI-GFP cells were a kind gift from Tokuko Haraguchi (Osaka University, Japan).

HeLa cells and HeLa lacI-GFP cells were cultured in full growth medium (DMEM, supplemented with FBS (10%), and penicillin-streptomycin (1%), or G418 (0.2 mg/mL), respectively, at typical mammalian cell culture conditions (37°C, 5% CO_2_, humidified atmosphere). The cells were used for experiments in passage number 5–30 and tested negative for mycoplasma contamination (MycoAlert Kit, Lonza AG). In preparation for a transfection experiment the cells were trypsinized and seeded to reach 50% confluency on the day of transfection.

**Transfection**

Medium was replaced with fresh medium 30 min prior transfection of the cells. Particle formation with X-tremeGENE^TM^ 9 was performed according to the manufacturer’s protocol with a 3:1 ratio of transfection reagent volume to DNA mass. After 15-20 min of particle formation, the transfection agent-DNA complex was given to the cells in full growth medium and incubated for 17-24 h.

**Confocal laser microscopy**

Confocal laser microscopy was performed on an Eclipse Ti2 inverse spinning disk confocal microscope (Nikon) using an 100x 1.45 CFI Plan Apo Oil objective (Nikon) and a sCMOS Orca Fusion BT camera (2304 x 2304 pixel size, Hamamatsu). The images were processed and analyzed with the software ImageJ.

**Living cells sample preparation and imaging (Figure 4)**

HeLa cells were seeded in an 8 well slide. Cells at 50% confluency were transfected with 0.5 µg Cy3-labeled pDNA (Mirus bio) as described above. After 17 h incubation with the transfection agent-DNA complex under standard incubation conditions, the cells were washed three times with 37°C PBS. Cells were incubated simultaneously with Hoechst 33342 (2.5 µg/mL) and CellMask™ Deep Red (0.625 µg/mL) in PBS for 10 min at 37°C. Subsequently, cells were washed three times with 37°C PBS and immediately imaged in Live Cell Imaging Solution. Sample excitation on the confocal microscope was performed at 405, 561, and 647 nm with illumination intensities of 40%, 20%, and 1%, respectively. 27 z‐stacks were acquired with a slice‐thickness of 0.2 µm.

**Fixed cell sample preparation and imaging (Figure 7)**

PlacO-RFP (Addgene plasmid #179507) was amplified in chemically competent E. coli DH5α cells obtained from Promega AG and purified via QIAprep Spin Miniprep Kit (Qiagen). HeLa lacI-GFP cells were seeded on cover glasses in a 24-well plate. Cells at 50% confluency were transfected with 0.5 µg placO-RFP. After 24 h incubation with the transfection agent-DNA complex under standard incubation conditions, the coverslips were washed three times with 37 °C PBS. Then, the cells were fixed with paraformaldehyde solution (4%) in PBS for 10 min at room temperature. The paraformaldehyde was discarded and the coverslips were washed three times with PBS. Subsequently, the cells were incubated with Hoechst 33342 (2.5 µg/mL) in PBS for 10 min at room temperature. The coverslips were again washed three times with PBS and mounted on glass specimen with 7 µL of Mowiol 4–88. The sample was imaged one day after sample preparation. Sample excitation on the confocal microscope was performed at 405, 488, and 561 nm with illumination intensities of 8%, 20%, and 50%, or 30%, 50%, and 15% for untransfected cells, respectively. Displayed intensity values are similar for both image files (110 to 500 for GFP).
